# Nonradiology Health Care Professionals Significantly Benefit From AI Assistance in Emergency-Related Chest Radiography Interpretation

**DOI:** 10.1016/j.chest.2024.01.039

**Published:** 2024-01-29

**Authors:** Jan Rudolph, Christian Huemmer, Alexander Preuhs, Giulia Buizza, Boj F. Hoppe, Julien Dinkel, Vanessa Koliogiannis, Nicola Fink, Sophia S. Goller, Vincent Schwarze, Nabeel Mansour, Vanessa F. Schmidt, Maximilian Fischer, Maximilian Jörgens, Najib Ben Khaled, Thomas Liebig, Jens Ricke, Johannes Rueckel, Bastian O. Sabel

**Affiliations:** a Department of Radiology, University Hospital, LMU Munich, Munich, Germany; b Institute of Neuroradiology, University Hospital, LMU Munich, Munich, Germany; c XP Technology and Innovation, Siemens Healthcare GmbH, Forchheim, Germany; d Department of Medicine I, University Hospital, LMU Munich, Munich, Germany; e Department of Medicine II, University Hospital, LMU Munich, Munich, Germany; f Department of Orthopaedics and Trauma Surgery, Musculoskeletal University Center Munich (MUM), University Hospital, LMU Munich, Munich, Germany; g Comprehensive Pneumology Center, German Center for Lung Research, Munich, Germany; h Department of Radiology, Asklepios Fachklinik München, Gauting, Germany

**Keywords:** AI assistance, artificial intelligence, chest radiography, emergency unit

## Abstract

**Background:**

Chest radiographs (CXRs) are still of crucial importance in primary diagnostics, but their interpretation poses difficulties at times.

**Research Question:**

Can a convolutional neural network-based artificial intelligence (AI) system that interprets CXRs add value in an emergency unit setting?

**Study Design and Methods:**

A total of 563 CXRs acquired in the emergency unit of a major university hospital were retrospectively assessed twice by three board-certified radiologists, three radiology residents, and three emergency unit-experienced nonradiology residents (NRRs). They used a two-step reading process: (1) without AI support; and (2) with AI support providing additional images with AI overlays. Suspicion of four suspected pathologies (pleural effusion, pneumothorax, consolidations suspicious for pneumonia, and nodules) was reported on a five-point confidence scale. Confidence scores of the board-certified radiologists were converted into four binary reference standards of different sensitivities. Performance by radiology residents and NRRs without AI support/with AI support were statistically compared by using receiver-operating characteristics (ROCs), Youden statistics, and operating point metrics derived from fitted ROC curves.

**Results:**

NRRs could significantly improve performance, sensitivity, and accuracy with AI support in all four pathologies tested. In the most sensitive reference standard (reference standard IV), NRR consensus improved the area under the ROC curve (mean, 95% CI) in the detection of the time-critical pathology pneumothorax from 0.846 (0.785-0.907) without AI support to 0.974 (0.947-1.000) with AI support (*P* < .001), which represented a gain of 30% in sensitivity and 2% in accuracy (while maintaining an optimized specificity). The most pronounced effect was observed in nodule detection, with NRR with AI support improving sensitivity by 53% and accuracy by 7% (area under the ROC curve without AI support, 0.723 [0.661-0.785]; with AI support, 0.890 [0.848-0.931]; *P* < .001). Radiology residents had smaller, mostly nonsignificant gains in performance, sensitivity, and accuracy with AI support.

**Interpretation:**

We found that in an emergency unit setting without 24/7 radiology coverage, the presented AI solution features an excellent clinical support tool to nonradiologists, similar to a second reader, and allows for a more accurate primary diagnosis and thus earlier therapy initiation.

**Take-home Points**

**Research Question:** Can a chest radiograph-interpreting artificial intelligence tool add value in an emergency unit setting?

**Results:** In all four pathologies (pneumothorax, pleural effusion, consolidation with suspected pneumonia, and nodules) that were tested on an emergency unit-derived cohort with 563 chest radiographs, nonradiology residents significantly improved their performance with AI support (*P* < .001 for all pathologies) and were able to improve their sensitivity and accuracy for all pathologies while maintaining the same (optimized) specificity.

**Interpretation:** The examined artificial intelligence tool has the potential to effectively assist nonradiologists in routine clinical practice within the emergency unit, serving as a “second reader.” This could lead to more accurate initial diagnostics and enhanced patient care by possibly initiating therapy at earlier stages.

Chest radiographs (CXRs) with typical indications such as suspected pneumonia, pneumothorax, pleural effusion, nodules, or catheter position checks remain a key tool in primary diagnostics, with a vast number of images ordered globally every day and a substantial impact on public health.^1^, ^2^, ^3^, ^4^, ^5^ Especially in the emergency unit, the CXR often serves as an initial assessment of whether a disease is acute and thus requires immediate treatment (eg, pneumothorax or pneumonia). However, interpretation is not always straightforward; in particular, projection phenomena, superimpositions, and similar representations of different findings can complicate the assessment, which is why a high level of expertise is usually required for accurate evaluation.^6^, ^7^, ^8^ This issue primarily affects nonradiologists who do not regularly interpret diagnostic imaging but are required to make clinical decisions based on image findings in emergency units without 24/7 radiology coverage or in case of long reporting times.^9^

Recently, several artificial intelligence (AI) algorithms have shown the potential to match or surpass health care professionals, including many CXR-interpreting algorithms.^10^, ^11^, ^12^, ^13^, ^14^, ^15^, ^16^, ^17^ These solutions offer the potential not just to decrease missed findings but also to streamline workflows, possibly enhancing patient care.^18^, ^19^, ^20^, ^21^, ^22^ The majority of common CXR-interpreting algorithms have been primarily trained on public data using natural language processing and have been validated on a subset of these data sets.^23^, ^24^, ^25^, ^26^ In previous studies, we have shown that public data have limitations, manual annotations significantly improve performance, and external validation is essential.^27^, ^28^, ^29^

The current study evaluated a CXR-interpreting AI algorithm that was trained on both public and expert-labeled data from multiple hospitals, detecting consolidations suspicious for pneumonia, pneumothorax, nodules, and pleural effusions. This is a follow-up study to a clinically oriented validation study comparing the performance of the algorithm in an emergency unit scenario with that of radiology residents (RRs) and nonradiology residents (NRRs) using consensus reading by three board-certified radiologists (BCRs) as the gold standard.^30^ The current study delves deeper by incorporating an additional AI-assisted reading by the same nine readers following a washout period of approximately 12 months. In doing so, the gains in performance, sensitivity, and accuracy associated with the provided AI assistance can be accurately quantified. Based on our initial study results, we anticipate that the AI tool could be of particular interest to nonradiologists in an emergency unit setting without 24/7 radiology coverage.

## Study Design and Methods

Approval of the institutional ethics committee was obtained for this study (approval number 19-541). Informed consent was waived due to the retrospective character of the study.

### Data Selection and Image Annotation

The study cohort used included a total of 563 CXRs, all acquired in the emergency units of a large university/primary care hospital (University Hospital of LMU Munich). Rudolph et al^30^^,^^31^ provide a detailed description of the study cohort, including a flowchart of enrollment. Included cases were identified by a full-text search based on radiology reports from 2000 to 2018. The inclusion criteria were: presentation to one of the hospital’s emergency units (multiple sites), patient age ≥ 21 years (legal age in the United States), and the presence of posterior-anterior projection in upright positioning. Through preselection by an experienced RR, a data set was created that represents common findings from the emergency unit with a balanced prevalence (approximately 10%-20% each). The collective includes the following findings: images without suspected pathologies, pleural effusions, pneumothoraces, consolidations suspicious for pneumonia, and nodules. These pathologies cover a significant portion of acutely relevant and/or easily overlooked findings.^32^ Of these four pathologies, malignant neoplasms of the lung and pneumonia are among the top five respiratory diseases in terms of global burden.^33^

Posterior-anterior projections of identified CXRs were exported as DICOM files and anonymized. Data were analyzed in a two-stage reading study by a total of nine readers: three BCRs (17, 9, and 7 years of experience, respectively, in thoracic imaging at the time of the initial reading study [constituted the reference standard (RFS)]), three RRs (4, 3, and 2 years of experience; the RR who performed the preselection did not participate in the reading), and three emergency unit-experienced NRRs (from cardiology [4 years of emergency unit experience at baseline], gastroenterology [3 years of emergency unit experience], and traumatology [1 year of emergency unit experience]).

In the first reading (Reading I), the 563 CXRs were evaluated by all readers regarding the aforementioned pathologies. The evaluation was performed on a 5-point Likert scale: 0, no suspicion; 1, unlikely; 2, possible; 3, likely; and 4, safe presence. Regarding nodules, readers were primarily directed to assess any nodule of diverse entities, including benign granulomas, a category we denote as “simple nodule detection” in subsequent text. If a nodule was identified (Likert scale > 0), readers were also prompted to assess whether they deemed an additional CT scan to be necessary. Documentation was conducted using spreadsheets, wherein each reader was required to assign a confidence score on the Likert scale individually to each pathology for both the right and left hemithorax in every case. The final score assigned for that pathology was the higher of the scores from the right/left hemithorax evaluations. The corresponding results have already been published (with a focus of AI validation^29^^,^^30^ and interobserver agreement^31^).

**Figure 1:**
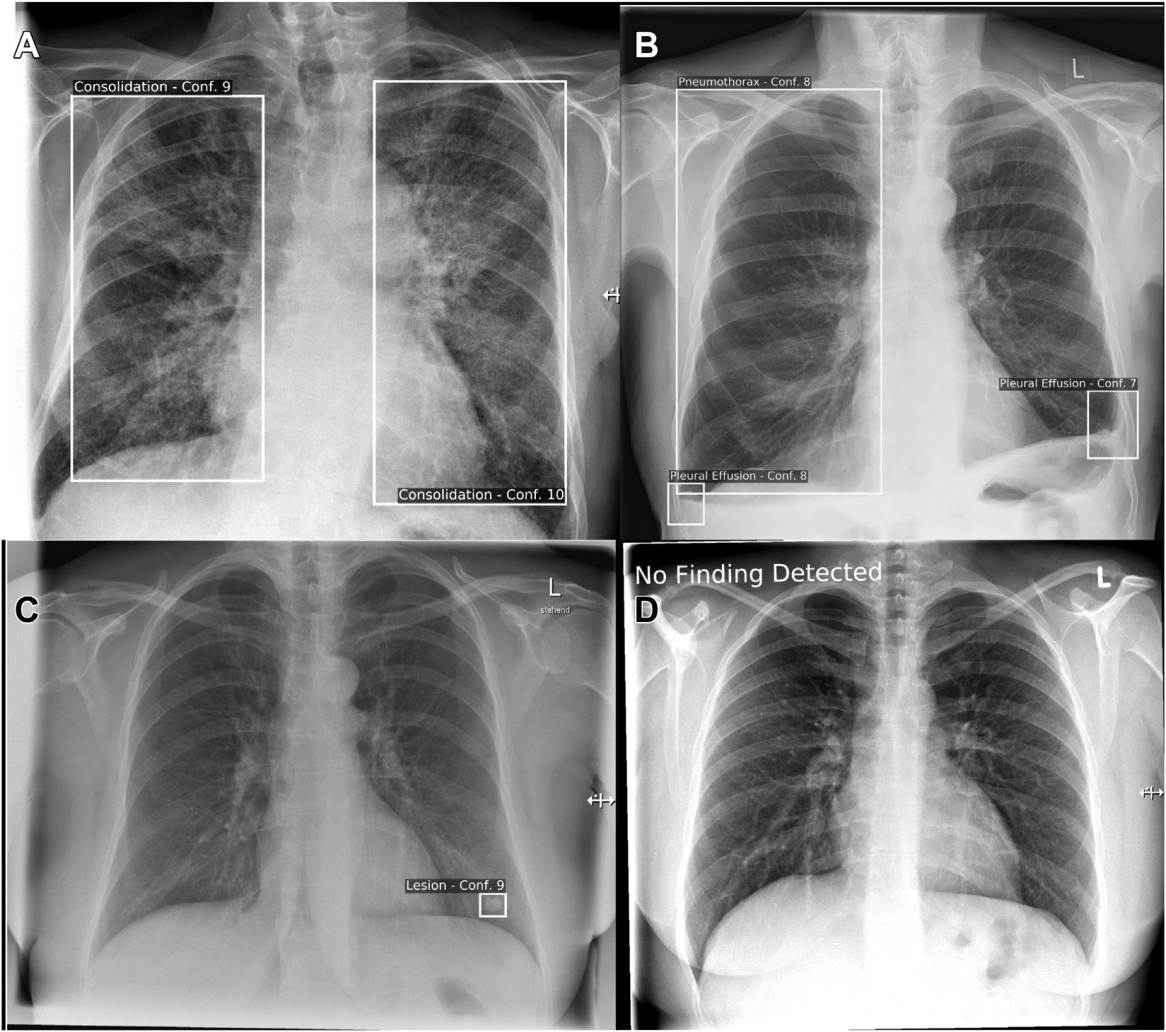
A-D, Examples of artificial intelligence secondary captures. A, Correctly identified bilateral consolidations that are suspicious for pneumonia considering the depth of inspiration. B, Both the right-sided seropneumothorax with basal air-fluid level as well as the shadowing in the left costodiaphragmatic recess (possible pleural effusion or pleural fibrosis) were identified. C, The solitary nodule in the left lower lung was correctly identified. D, In case the artificial intelligence algorithm did not identify any of the four pathologies on the chest radiograph, the image was marked as “No Finding Detected.”

The second reading took place after a washout period of approximately 12 months. In Reading II, the same readers re-assessed the same images with AI support (wAI) according to the same methodology mentioned earlier. Readers were instructed not to access their ratings from Reading I. The AI support included a visual representation of the algorithm results in the form of overlays on secondary captures with AI confidence values of 4 to 10 (4 = low confidence, 10 = high confidence) (Fig 1). Neither the readers (in both Reading I and Reading II) nor the AI algorithm had access to preliminary CXR examinations or clinical parameters.

### AI Algorithm

The AI algorithm (including the version/release) corresponds to the one used from the first validation study^30^ and is described as follows. The chosen network is a single-shot object detection network that consists of a residual network-based backbone followed by a convolutional feature pyramid network. Then, based on the respective pyramid layers, a predictor network estimates the respective class probabilities on different scales. Input images are resized to a shape of 1,025 × 1,025 by applying bilinear interpolation and preserving the original aspect ratio. The resulting pixel values are transformed by using a robust intensity normalization technique. Finally, image augmentation is applied using left/right image flip, random cropping and scaling, random rotations, and (inverse) gamma transforms. The detection system for pneumothorax, nodule, consolidation, and pleural effusion is trained in a multiclass setting by jointly classifying and detecting these abnormalities. The feature extractor generates candidates in an abstract feature space that are consumed by the discriminator subnetwork to compute probabilities of abnormality existence in the image subregion of interest. A graphic illustration can be found in Figure 2 from Rudolph et al.^30^ This fully convolutional architecture processes the entire image in a single shot while analyzing its content on multiple scales to capture both global and local comorbidities present inside the image. The network is trained by optimizing a threefold loss term, consisting of: (1) a classification loss based on the focal loss^34^; (2) a coordinate regression loss based on the overlap of bounding boxes; and (3) a bounding box-based centerness loss, which is based on weighted binary cross entropy.

Training data cover acquisitions from all main vendors and contain images from 18 sites distributed throughout Europe, Asia, North America, and South America. Data were extracted based on sequential sampling and preselected by applying natural language processing to associated radiology reports (if available). This strategy is essential to identify low-prevalent cases with pleural effusion, pneumothorax, consolidation, or nodule. To train the algorithm as described earlier, an RFS that includes location information encoded as tight boxes around the respective abnormality as well as the abnormality label is required. This is established by a majority voting of BCRs in a multistage setup. For each RFS to be as robust as possible, each radiologist is trained on the task using predefined training material, including detailed annotation specifications as well as tool training. In addition to the structured annotation process, data are reviewed regularly and, if necessary, updated. More details on the data distribution of different pathologies are shown in Table 1.

**Table 1:** Training Data in the Different Pathologies

**Pathology**	Training (Total/Positives/Negatives)	Validation (Total/Positives/Negatives)	Internal Validation (AUC)
Pneumothorax (AP + PA)	11,260/1,068/10,192	318/67/251	0.980
Pleural effusion (PA)	10,276/2,042/8,234	332/74/258	0.995
Consolidation suspicious of pneumonia (AP + PA)	11,622/5,653/5,969	540/261/279	0.960
Nodules (PA)	9,784/4,986/4,798	444/138/306	0.950

Note that the training data did not change compared with the first study because the same algorithm was used.^30^ AP = anterior-posterior; AUC = area under the receiver-operating characteristic curve; PA = posterior-anterior.

### Image Analysis, Results Quantification, and Statistical Analysis

The performances of the AI algorithm and the RR and NRR readers (without AI support [woAI] and with AI support [wAI]) were quantified by receiver-operating characteristic (ROC) analysis and calculation of the area under the ROC curve (AUC) with CIs and the DeLong test for AUC woAI/wAI comparison (R package “pROC,” function “ci,” and “roc.test” method “DeLong”^35^^,^^36^). The BCR’s reading served as the gold standard.

To form RFSs that allow a yes-or-no call but also reflect the diagnostic uncertainty of the readers, individual Likert scale evaluations were pooled as follows. In the very specific RFS I, scores 0 to 3 are scored as negative and only 4 as positive; in the very sensitive RFS IV, scores 1 to 4 are scored as positive and only 0 as negative (Table 2). The intermediate RFS II/III are formed as described in Table 2. The final RFSs (RFS I-IV), which take into account the readings of all three BCRs, were determined based on the principle of majority voting. The results from the individual BCR readings were thus combined into composite assessments. We incorporated the BCRs’ evaluations derived from the AI-assisted reading as we believe that this approach ensures the highest possible diagnostic accuracy. Artificial RR/NRR consensus was formed by summing up the individual scores of the RRs/NRRs, finally leading to theoretically 15-level confidence scores (3 × 5).

**Table 2:** Tabular Representation of the Formation of RFS I to IV Based on Readings of the BCRs

Reference Standard	Score 0 “No Suspicion”	Score 1 “Unlikely”	Score 2 “Possible”	Score 3 “Likely”	Score 4 “Safe Presence”
RFS I	Negative	Negative	Negative	Negative	Positive
RFS II	Negative	Negative	Negative	Positive	Positive
RFS III	Negative	Negative	Positive	Positive	Positive
RFS IV	Negative	Positive	Positive	Positive	Positive

The 5-level Likert-based confidence scores were divided into binary yes-or-no calls of different sensitivity for each BCR. A consensus was formed from the three readings, representing the final RFSs. This procedure was applied pathology-specifically to all BCR readings and all CXRs. BCR = board-certified radiologist; CXR = chest radiograph; RFS = reference standard.

ROC curves of the woAI reading were used to approximate operating points to the maximal sum of sensitivity and specificity according to Youden J statistics.^37^ The resulting diagnostic metrics for the optimized operating points (accuracy, sensitivity, specificity, positive and negative predictive values, false-positive rate, and false-negative rate) were calculated. To compare ROC curves of both readings, RR/NRR consensus ROCs (smoothened by the more finely graded Likert scale as mentioned earlier) were used for a further statistical ROC curve fitting by applying a linear model fitting to the quantiles of the sensitivities and specificities (R package “pROC,” function “smooth,” method “binormal”^35^). Continuously extractable operating points on the fitted curves allowed to quantify the AI-associated gain in sensitivity/accuracy, for example, while maintaining a fixed specificity. This fixed specificity by definition was derived again from Youden statistics applied to the fitted ROC curve of the non-AI-assisted reading. All statistical calculations and visual illustrations were performed by using the open-source programming language R.^38^

## Results

The values for AUC, sensitivity, and accuracy given here assume values between 0 and 1, with values close to 1 indicating better performance/sensitivity/accuracy. *P* values < .05 are considered statistically significant.

### Pneumothorax

**Figure 2:**
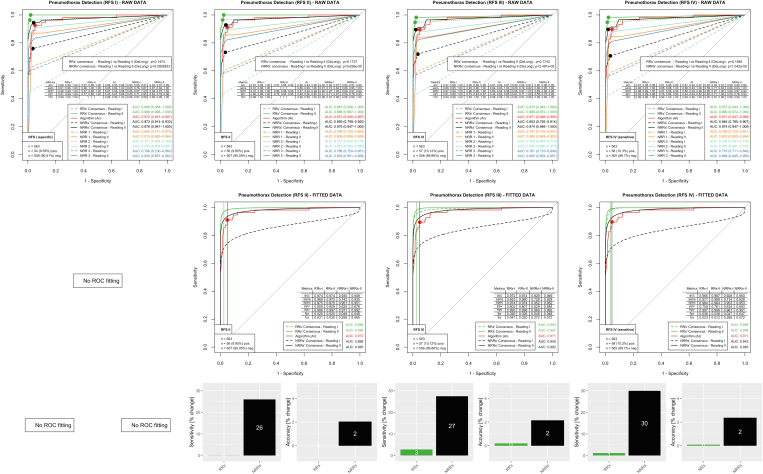
Evaluation for pathology pneumothorax. The first row shows performance analyses for all different board-certified radiologists’ reading-based RFS (RFS I-IV, from the very specific reading in RFS I to the very sensitive reading in RFS IV) considering the RAW data (no curve fitting). ROC curves of RR and NRR consensus calculated from the sums of the reading scores, the curves of the NRR individual readings (NRR1, NRR2, and NRR3) and the AI algorithm are plotted in each diagram. For the human readers, the dashed lines represent without AI support Reading I and the continuous lines with AI support Reading II. The points marked on the ROC curves represent the operating points optimized according to Youden statistics, to which the metrics shown there also refer. AUC values are shown in the lower right corner with 95% CIs. The second row shows FITTED ROC data. RR/NRR consensus raw data were used for a statistical ROC curve fitting. For RFS I, statistical ROC curve fitting failed due to an NRR/RR consensus raw data ROC providing too few data points. Operating points on the woAI ROC curves (Reading 1, dashed lines) were approximated according to Youden statistics, and the resulting “iso-specificities” are illustrated by vertical lines. Operating points on the wAI ROC curves (Reading 2, continuous lines) were approximated to the intersections with the iso-specificity lines. The diagnostic operating point metrics are illustrated in tabular form on the bottom right**.** In the third row, barplots show the relative change in sensitivity and accuracy from the without AI support to the with AI support reading, based on the preserved iso-specificities. The evaluation shows that especially the NRR readers can improve significantly with AI support. In the clinically relevant RFS IV, the increase in sensitivity is 30% and the increase in accuracy is 2%. acc = accuracy; AI = artificial intelligence; AUC = area under the receiver-operating characteristic curve; fnr = false-negative rate; fpr = false-positive rate; npv = negative predictive value; NRR = nonradiology resident; ppv = positive predictive value; RFS = reference standards; ROC = receiver-operating characteristic; RR = radiology resident; sens = sensitivity; spec = specificity.

Figure 2 illustrates the assessment in the pathology pneumothorax, featuring ROC curves, fitted ROC curves, and barplots depicting alterations in sensitivity and accuracy (from woAI reading to wAI reading). RRs as well as each individual NRR benefited from AI assistance, which could be shown for all applied RFSs (RAW DATA). When considering the most sensitive RFS IV, the AI performed with an AUC of 0.971 (95% CI, 0.947-0.995), and RRs and NRRs benefited from AI assistance. The RR consensus (RAW DATA) already showed good performance woAI with an AUC of 0.973 (95% CI, 0.943-1.000) but tended to improve wAI to an AUC of 0.990 (95% CI, 0.972-1.000; *P* = .17). The NRR consensus performance (RAW DATA) significantly increased from a woAI AUC of 0.846 (95% CI, 0.785-0.907) to a wAI AUC of 0.974 (95% CI, 0.947-1.000; *P* < .001). Youden operating point optimization based on the fitted woAI ROC curves revealed specificities of 0.964 for the RR consensus and 0.953 for the NRR consensus.

By using the fitted wAI ROC curves for an operating point approximation to these preserved specificities, the associated sensitivities increased from 0.977 to 0.988 (1% increase) for the RR consensus and from 0.714 to 0.928 (30% increase) for the NRR consensus. Associated accuracies increased from 0.966 to 0.967 (0% increase, RR consensus) and from 0.928 to 0.950 (2% increase, NRR consensus), respectively. Thus, RRs with an excellent baseline performance only benefited slightly from AI assistance, but AI assistance considerably increased the pneumothorax detection sensitivity/accuracy of NRRs by 30%/2%.

### Pleural Effusion

**Figure 3:**
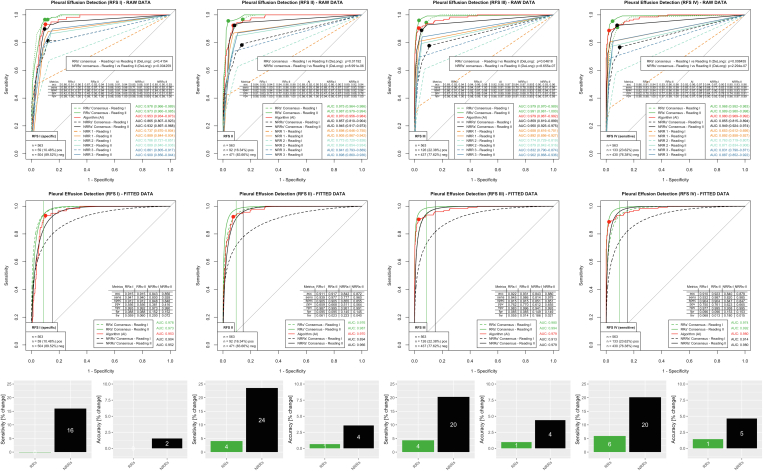
Evaluation for pathology pleural effusion. Performance analysis was performed analogously to the other pathologies (see caption of Figure 2). In the clinically relevant RFS IV (most sensitive), there is a considerable improvement of NRRs’ performance with a sensitivity increase of 20% and an accuracy increase of 5%. The RR also improved with AI support (sensitivity +6%, accuracy +1%). acc = accuracy; AI = artificial intelligence; AUC = area under the receiver-operating characteristic curve; fnr = false-negative rate; fpr = false-positive rate; npv = negative predictive value; NRR = nonradiology resident; ppv = positive predictive value; RFS = reference standards; RR = radiology resident; sens = sensitivity; spec = specificity.

The outcomes pertaining to pleural effusion are presented in Figure 3. RRs and all individual NRRs could benefit from AI assistance in almost all reference standards (RAW DATA, except RR consensus in RFS I [no significant change here]). AI showed an AUC of 0.980 (95% CI, 0.969-0.992) for the clinically very relevant RFS IV. RR consensus already performed well in woAI Reading with an AUC of 0.968 (95% CI, 0.952-0.983) and improved wAI to an AUC of 0.989 (95% CI, 0.980-0.998; *P* < .01). The NRR consensus improved from an AUC of 0.855 (95% CI, 0.815-0.894) woAI to 0.949 (95% CI, 0.924-0.974) wAI (*P* < .001). The operating point optimized on the fitted plots in the woAI Reading according to Youden operating point optimization showed a specificity of 0.904 for the RR consensus and 0.847 for the NRR consensus. Maintaining this specificity, the fitted wAI ROC curves showed an increase in sensitivity from 0.932 to 0.987 (6% increase) for the RR consensus and from 0.820 to 0.985 (20% increase) for the NRR consensus. Accuracy increased accordingly from 0.910 to 0.923 (1% increase, RR consensus) and from 0.840 to 0.879 (5% increase, NRR consensus). With already good performance woAI, RRs saw a slight improvement with AI support. NRRs, on the other hand, improved significantly, increasing their sensitivity by 20% and accuracy by 5%.

### Consolidations Suspicious for Pneumonia

**Figure 4:**
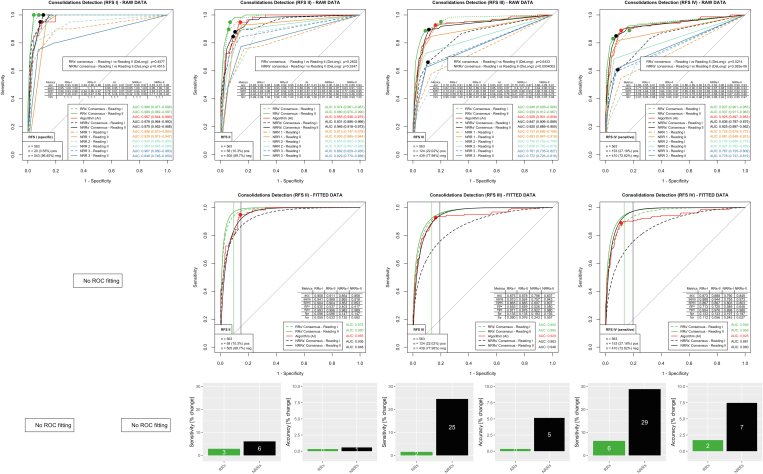
Evaluation for pathology consolidation suspicious for pneumonia. The analysis was performed similarly to the other pathologies (see caption of Figure 2). For RFS I, statistical ROC curve fitting failed because the NRR/RR consensus ROC raw data provided too small a number of data points. In the clinically relevant RFS IV, there is a substantial performance gain in the NRR consensus group with AI support (sensitivity, +29%; accuracy, +7%). The RRs also improved slightly with AI support (sensitivity, +6%; accuracy, +2%). acc = accuracy; AI = artificial intelligence; AUC = area under the receiver-operating characteristic curve; fnr = false-negative rate; fpr = false-positive rate; npv = negative predictive value; NRR = nonradiology resident; ppv = positive predictive value; RFS = reference standard; ROC = receiver-operating characteristic; RR = radiology resident; sens = sensitivity; spec = specificity.

Figure 4 shows the results for consolidations suspicious for pneumonia. RRs and all individual NRRs could benefit from AI assistance in all reference standards (RAW DATA). AI showed an AUC of 0.925 (95% CI, 0.897-0.953) for the clinically very relevant RFS IV. RR consensus had an excellent performance with an AUC of 0.927 (95% CI, 0.901-0.953) already in the first reading woAI and improved to an AUC of 0.937 (95% CI, 0.911-0.963) wAI (*P* = .52). The NRR consensus improved from an AUC of 0.836 (95% CI, 0.797-0.875) to an AUC of 0.925 (95% CI, 0.897-0.952; *P* < .001). The specificity optimized by Youden statistics in the woAI reading (FITTED DATA) was 0.867 for the RR consensus and 0.803 for the NRR consensus. Upon holding this specificity constant, the fitted wAI ROC curves showed an increase in sensitivity from 0.888 to 0.944 (6% increase) for the RR consensus and from 0.755 to 0.973 (29% increase) for the NRR consensus. Accuracy increased from 0.873 to 0.888 (2% increase) for the RR consensus and from 0.790 to 0.849 for the NRR consensus (7% increase). In summary, with a very good performance, RRs could marginally improve woAI with AI support. In contrast, NRRs improved significantly with AI support (increase in sensitivity, 29%; increase in accuracy, 7%)

### Nodules

**Figure 5:**
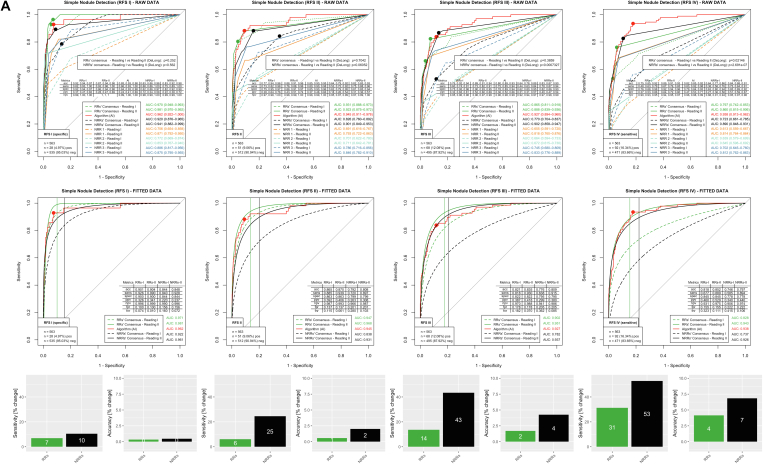

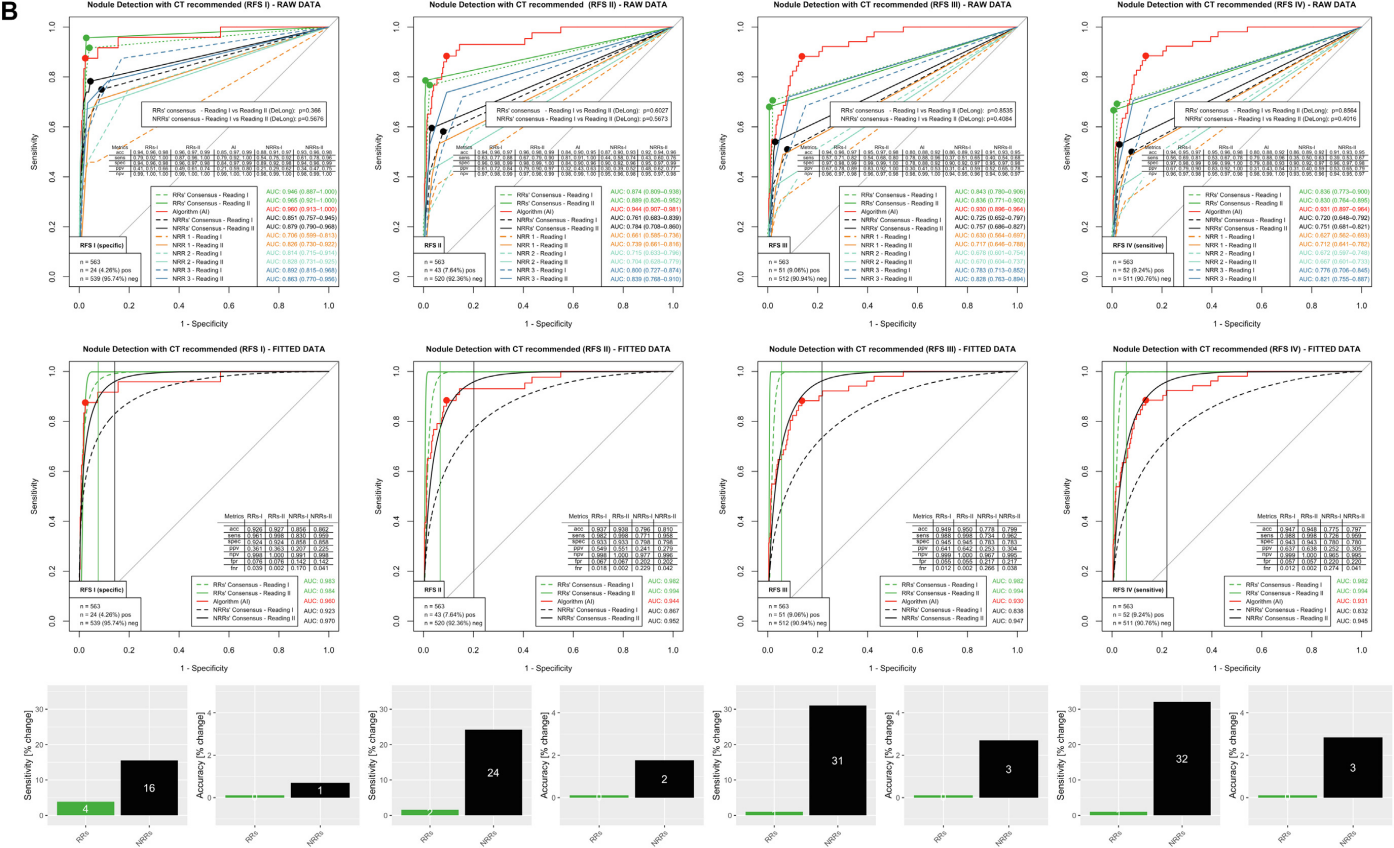
Evaluation for pathology nodules with (A) simple nodule detection only and (B) clinically relevant nodule detection for which an additional CT scan was recommended by the board-certified radiologists. Both RRs and NRRs were able to significantly increase their detection rate for nodules. In simple nodule detection in the clinically relevant RFS IV, the sensitivity increase in the RR consensus was 33% (with an accuracy increase of 4%) and in the NRR 53% (with an accuracy increase of 7%). Considering the clinically more relevant, potentially malignant nodules in RFS IV, NRR improved with AI support (sensitivity, +32%; accuracy, +3%). The RRs had similar performance values with AI support (sensitivity, +1%; accuracy, +/- 0%). acc = accuracy; AI = artificial intelligence; AUC = area under the receiver-operating characteristic curve; fnr = false-negative rate; fpr = false-positive rate; npv = negative predictive value; NRR = nonradiology resident; ppv = positive predictive value; RFS = reference standards; RR = radiology resident; sens = sensitivity; spec = specificity.

Results for nodules are provided in Figure 5. Both RRs and all individual NRRs were able to benefit from AI assistance in simple nodule detection in all reference standards (Fig 5A, RAW DATA). However, when considering the clinically relevant nodules in which BCRs indicated CT imaging for further assessment, RRs benefited only slightly, but NRRs benefited very significantly from AI support (Fig 5B, RAW DATA).

Regarding the simple nodule detection in the clinically relevant RFS IV, AI performed with an AUC of 0.938 (95% CI, 0.913-0.962), demonstrating the best performance of all ROC curves (Fig 5A). RR consensus improved its performance with AI assistance from an AUC of 0.797 (95% CI, 0.742-0.853) woAI to 0.860 (95% CI, 0.815-0.906) wAI (*P* < .05). For NRR consensus, the change was from 0.723 (95% CI, 0.661-0.785) woAI to 0.890 (95% CI, 0.848-0.931) wAI (*P* < .001). According to Youden statistics, optimized operating points on the fitted data of the woAI reading resulted in specificities of 0.845 for the RR consensus and 0.778 for the NRR consensus. When these specificities were kept constant, there was an increase in sensitivity from 0.677 to 0.889 (31% increase) for the RR consensus and from 0.585 to 0.894 (53% increase) for the NRR consensus on the fitted graphs wAI. Accuracy increased from 0.818 to 0.852 for the RR consensus (4% increase) and from 0.746 to 0.797 (7% increase) for the NRR consensus.

Concerning clinically relevant nodules with additional CT imaging recommended by the BCRs, AI showed the best performance of all ROC curves in the clinically relevant RFS IV with an AUC of 0.931 (95% CI, 0.897-0.964) (Fig 5B). RR consensus showed an AUC of 0.830 (95% CI, 0.764-0.895) woAI, comparable to 0.836 (95% CI, 0.773-0.900) in the second reading wAI (*P* = .86). NRR consensus improved from 0.720 (95% CI, 0.648-0.792) woAI to 0.751 (95% CI, 0.681-0.821) wAI (*P* = .40). Operating points based on fitted data and woAI reading yielded a specificity of 0.943 for RR consensus and 0.780 for NRR consensus. By maintaining these specificities, there was a sensitivity increase on the fitted ROC curves of the woAI reading from 0.988 to 0.998 (1% increase) for the RR consensus and from 0.726 to 0.959 (32% increase) for the NRR consensus. Correspondingly, accuracy changed from 0.947 to 0.948 (0% increase) for RR consensus and from 0.775 to 0.797 (3% increase) for NRR consensus.

In summary, RRs significantly improved their performance in simple nodule detection with AI support (increase in sensitivity, 31%; increase in accuracy, 4%) but did not significantly improve in the detection of clinically relevant nodules (for which further workup is required). The NRRs, however, improved significantly both in pure nodule detection (increase in sensitivity, 53%; increase in accuracy, 7%) and in the detection of clinically relevant nodules (increase in sensitivity, 32%; increase in accuracy, 3%).

## Discussion

The current study showed that AI support for lung pathology detection resulted in significant performance gains for NRR readers for all pathologies tested. Although preserved specificities (according to Youden optimization) remained the same, sensitivities and accuracies improved, in some cases dramatically, with AI support: The most significant effect was observed in the detection of nodules, with the NRR readers increasing their sensitivity by up to 53% and their accuracy by up to 7% wAI (RFS IV). The effects were less pronounced for the RRs, who displayed a high level of performance at baseline without AI support. In a prior study, we showed that this AI algorithm can mimic the performance level of RRs for most pathologies, and we postulated that NRRs might potentially benefit from the AI results.^30^ This assumption was quantitatively confirmed in the current study.

### Impact on Individual Reader Performance

Non-radiologists who may be uncertain about CXR diagnostics could find increased confidence by being aware of the enhanced performance achieved with AI support. This is particularly relevant when a radiology department lacks round-the-clock coverage or experiences prolonged reporting times due to an increased workload. In such cases, an AI algorithm could serve as a substitute for a radiologist, functioning as a technical “second reader.” Considering that night and weekend shifts are typically covered by proficient RRs, and given the comparable performance of the AI algorithm for the tested pathologies, there is potential for the algorithm to sustain this level of proficiency.

### Impact on Patient Care

From the patient's point of view, the improved performance enhances primary care during shift times. This is particularly crucial for time-critical pathologies requiring urgent treatment such as pneumothoraces. Considering that NRRs could enhance their sensitivity in detecting pneumothoraces by up to 30% (RFS IV), it is plausible that most NRRs missed relevant findings requiring urgent treatment without AI support. In addition to identifying pneumothorax, primary diagnostics play a crucial role in identifying pathologies such as consolidation suspicious for pneumonia and pleural effusion. A meticulous initial assessment can minimize the need for subsequent visits to the emergency unit and prevent inappropriate discharges. Early detection of nodules may indicate the presence of a primary lung tumor or a metastatic oncologic disease. Although these findings are usually not time critical, if they are the underlying cause of acute thoracic symptoms, they enable further clinical triage of the patient and can alleviate the burden on the emergency unit.

### Impact on the Daily Work of Radiologists

From the radiologist’s perspective, an NRR-enhancing AI algorithm can reduce the daily workload. It is conceivable, for example, that a radiologist on call will be called significantly less often for “trivial” findings and can therefore focus on more complex findings. One potential downside of AI support for less experienced readers might stem from an uncritical acceptance of results. For instance, they might blindly trust the AI outcomes due to uncertainties, potentially leading to an increase in false-positive results. In this regard, it is also important to ensure that standard diagnostic training is not compromised and that ethical dimensions of AI deployment are considered.^39^^,^^40^ However, a previous study has shown that physicians using a clinically implemented AI solution in an emergency unit scenario would be more likely not to blindly trust the results and that the introduction of AI into routine clinical practice has implications for perceptions and knowledge of AI applications.^41^

### Strengths and Limitations of the Study Design

The current study is particularly notable for its large number of readers and cases. Three BCRs, including a designated expert in thoracic radiology, provide a high-quality RFS. The RFS was further subdivided according to different sensitivities/specificities, reflecting natural uncertainties in the interpretation of CXRs. With a washout period of approximately 12 months and precise reading instructions, any major influence of the woAI reading on the wAI reading could be largely excluded. The algorithm used stands out due its extensive training data sourced from various centers. Biases in the detection of pneumothoraces identified in preliminary studies could be eliminated by further training on specially annotated data sets.^28^ The prototypical algorithm used in this study has been integrated into a commercially available product, ready to use and offers full Picture Archiving and Communication System integration based on a solution available in the cloud and on-premise.

Limitations of the study include a single-center evaluation with a pathology-enriched cohort, which does not reflect a true real-world scenario, a limited number of pathologies, inaccuracies arising from the statistical approximations used in plotting Likert-scaled objects (eg, interpolation of ROC curves, approximation of “fitted ROC curves”), and a natural training effect that occurred between Reading I and II that may also have led to performance improvement. Due to the commercial nature of the used algorithm, information about annotations or radiologists involved in the training cannot be disclosed. In addition, we used the latest algorithm version that was available at the time this study was conducted; however, newer versions of the algorithm are now available. Consequently, future availability of this algorithm version cannot be guaranteed.

## Interpretation

Physicians, particularly less experienced ones, can benefit from AI assistance in an emergency unit setting. The nonradiologists were able to increase their sensitivity and accuracy in all tested pathologies. This has a high clinical relevance, especially when 24/7 coverage by a radiology department and/or continuous support by experienced senior physicians cannot be guaranteed. In this case, the number of potentially missed findings could be reduced.

## Funding/Support

The Department of Radiology, University Hospital, LMU Munich received funding (research cooperation) from Siemens Healthineers.

## Financial/Nonfinancial Disclosures

The authors have reported to *CHEST* the following: B. O. S. and J. Rueckel received financial compensation for speaker's activities by Siemens Healthineers (lectures at conferences). C. H., A. P., and G. B. received financial compensation by Siemens Healthineers (employees). None declared (J. Rudolph, B. F. H., J. D., V. K., N. F., S. S. G., V. S., N. M., V. F. S., M. F., M. J., N. B. K., T. L., J. Ricke).
